# Supplementary material for: JMJD6 participates in the maintenance of ribosomal DNA integrity in response to DNA damage
Source: PLoS Genet. 2020 Jun 29;16(6):e1008511. doi: 10.1371/journal.pgen.1008511 (PMC7351224; doi:10.1371/journal.pgen.1008511)
Supplement: S1 Fig — (PDF) [file pgen.1008511.s001.pdf]

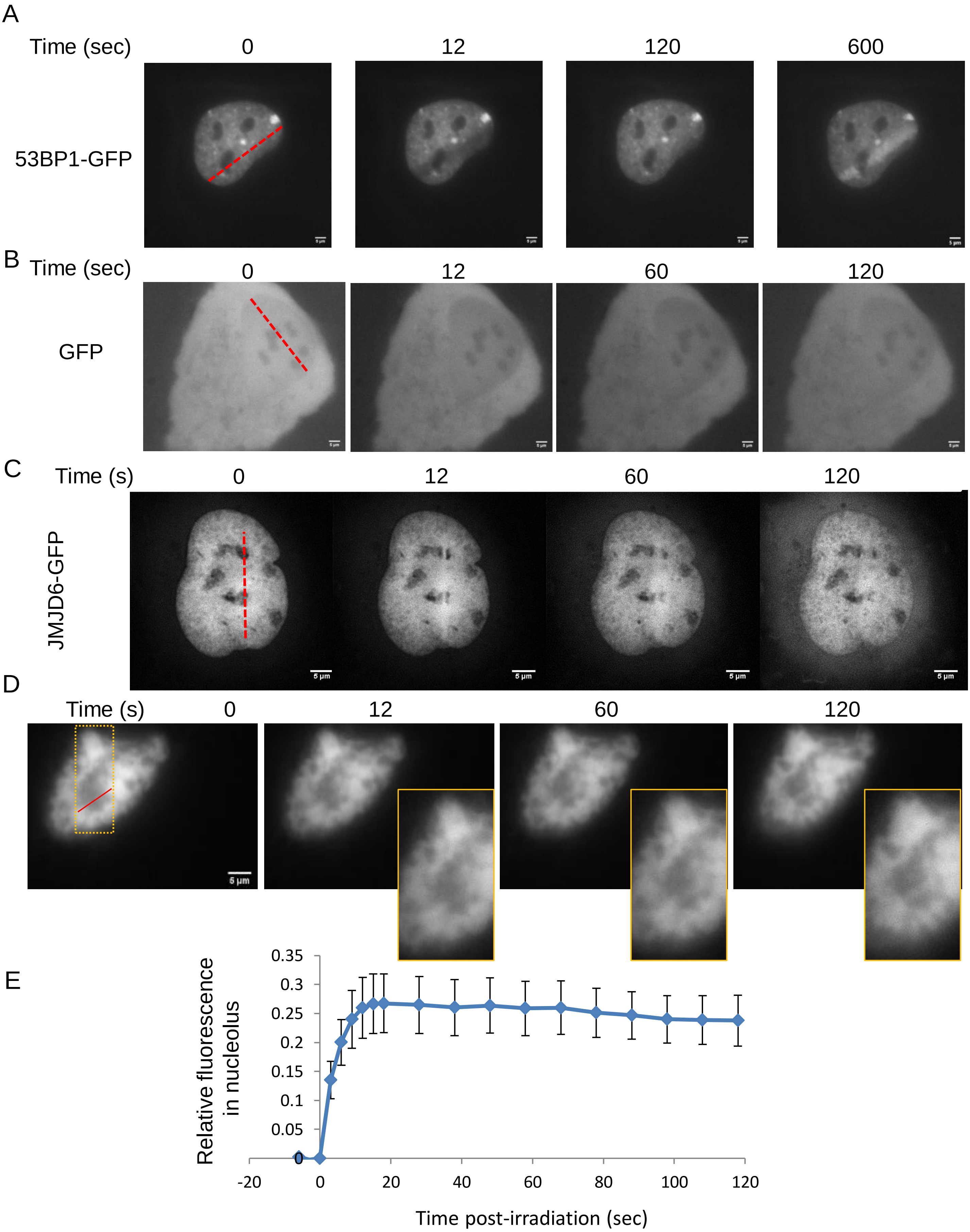

Fig S1. Laser induced DNA damage on living cells.

A. U2OS cells expressing 53BP1-GFP. Dotted red line indicated laser irradiation. The time post irradiation is indicated above images. B same as in A with cells expressing GFP protein. C. additional cells expressing JMJD6-GFP as in Figure 1. D. MRC5 cells expressing JMJD6-GFP. E. Kinetic of JMJD6-GFP accumulation in nucleolus of U2OS cells. Results are the mean +/- SEM of 10 cells.
