# Supplementary material for: JMJD6 participates in the maintenance of ribosomal DNA integrity in response to DNA damage
Source: PLoS Genet. 2020 Jun 29;16(6):e1008511. doi: 10.1371/journal.pgen.1008511 (PMC7351224; doi:10.1371/journal.pgen.1008511)
Supplement: S2 Fig — (PDF) [file pgen.1008511.s002.pdf]

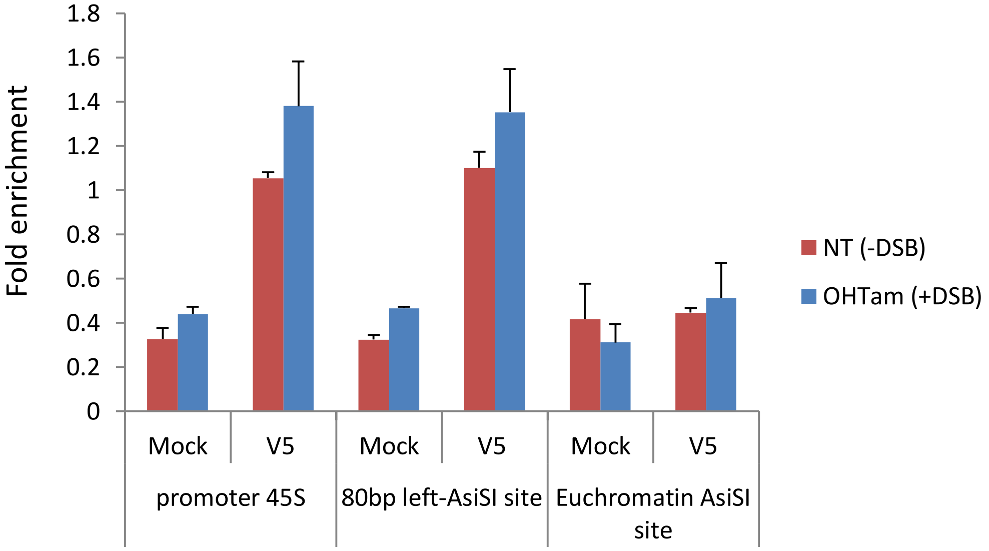


**Fig S2. JMJD6 recruitment at DSB assessed by ChIP**

Chromatin immunoprecipitation (ChIP) in DiVA cell line transfected with tagged JMJD6-V5. ChIP results are expressed as fold enrichment compared with signal obtained on beta-actin set at 1. Mock: no antibody; V5: anti V5 antibody. Results are expressed as mean+/- sd of triplicate qPCR.
