# Supplementary material for: JMJD6 participates in the maintenance of ribosomal DNA integrity in response to DNA damage
Source: PLoS Genet. 2020 Jun 29;16(6):e1008511. doi: 10.1371/journal.pgen.1008511 (PMC7351224; doi:10.1371/journal.pgen.1008511)
Supplement: S3 Fig — (PDF) [file pgen.1008511.s003.pdf]

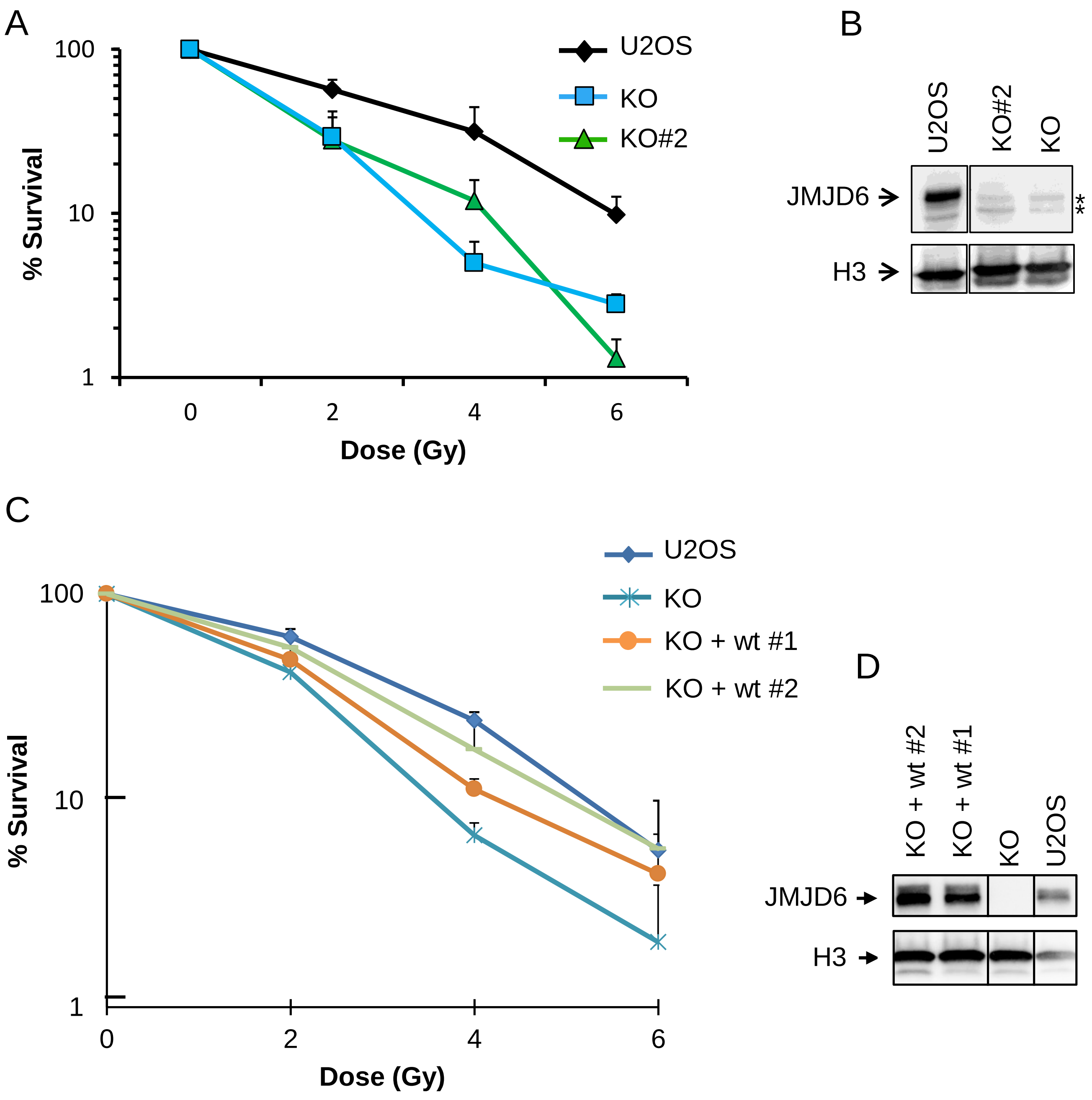

Fig S3: JMJD6 KO cell lines present increased sensitivity to ionizing radiations.
A. U2OS cells or two clones of U2OS cells inactivated for JMJD6 expression (KO and KO#2) were exposed to ionizing radiations and subjected to a clonogenic assay. The mean and standard deviation from three independent experiments are shown. B. Western blot analysis for JMJD6 expression using JMJD6 and Histone H3 antibodies. The bar indicates that the original image was cut. The stars indicate two non specific bands detected by the anti-JMJD6 antibody. C. same as in A with JMJD6-KO cell line complemented with JMJD6. D same as in B.
