# Supplementary material for: JMJD6 participates in the maintenance of ribosomal DNA integrity in response to DNA damage
Source: PLoS Genet. 2020 Jun 29;16(6):e1008511. doi: 10.1371/journal.pgen.1008511 (PMC7351224; doi:10.1371/journal.pgen.1008511)
Supplement: S4 Fig — (PDF) [file pgen.1008511.s004.pdf]

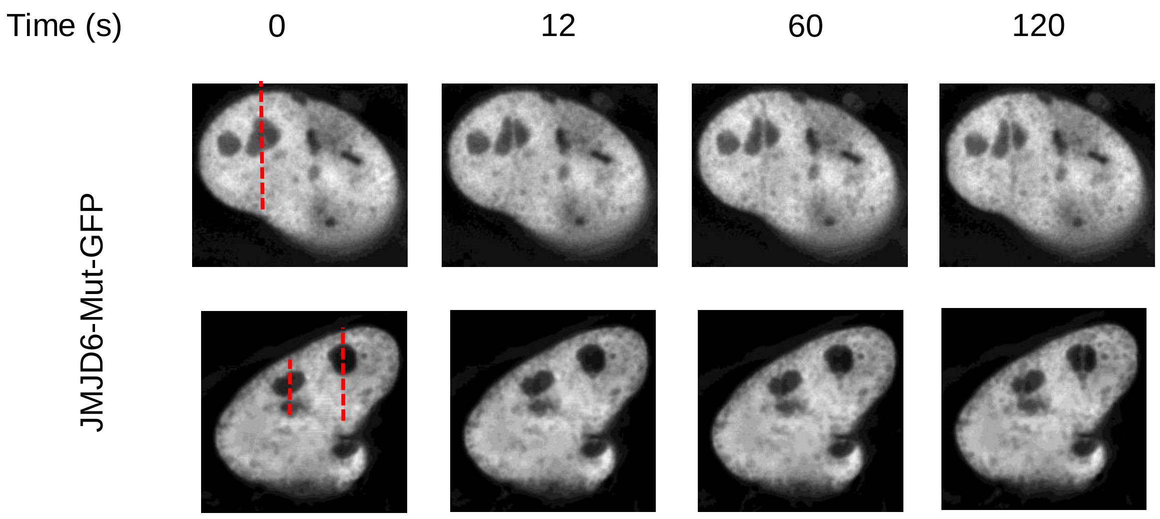

FigS4. Recruitment of JMJD6 Mutant at DNA damages.
U2OS cells expressing the inactive JMJD6-Mut-GFP. Dotted red line indicated laser irradiation. The time post irradiation is indicated above images.
