# Supplementary material for: JMJD6 participates in the maintenance of ribosomal DNA integrity in response to DNA damage
Source: PLoS Genet. 2020 Jun 29;16(6):e1008511. doi: 10.1371/journal.pgen.1008511 (PMC7351224; doi:10.1371/journal.pgen.1008511)
Supplement: S5 Fig — (PDF) [file pgen.1008511.s005.pdf]

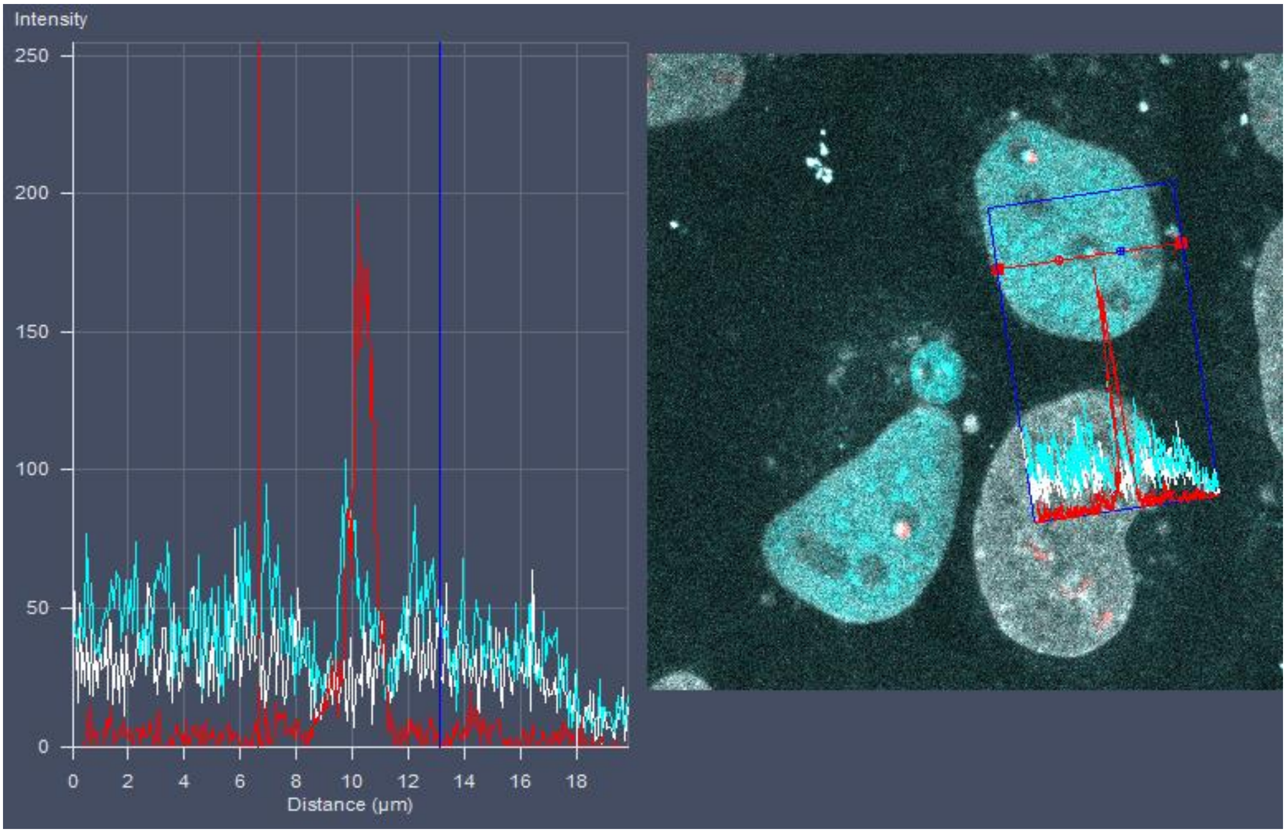

FigS5. JMJD6 colocalizes with Treacle in nucleolus.
(Right) Image of U2OS cells expressing V5-tagged-JMJD6 exposed to ionizing radiations at 5 Gy (1 h post-IR) analysed by confocal microscopy using anti-V5 and anti-Treacle antibodies. Left) Confocal line profile showing fluorescence intensity for Treacle (red), JMJD6-V5 (Cyan), and DAPI (white). Note the correspondence of the peaks of Treacle and JMJD6 in DAPI low region corresponding to the nucleolus (between the red and blue vertical lines).
