# Supplementary material for: JMJD6 participates in the maintenance of ribosomal DNA integrity in response to DNA damage
Source: PLoS Genet. 2020 Jun 29;16(6):e1008511. doi: 10.1371/journal.pgen.1008511 (PMC7351224; doi:10.1371/journal.pgen.1008511)
Supplement: S6 Fig — (PDF) [file pgen.1008511.s006.pdf]

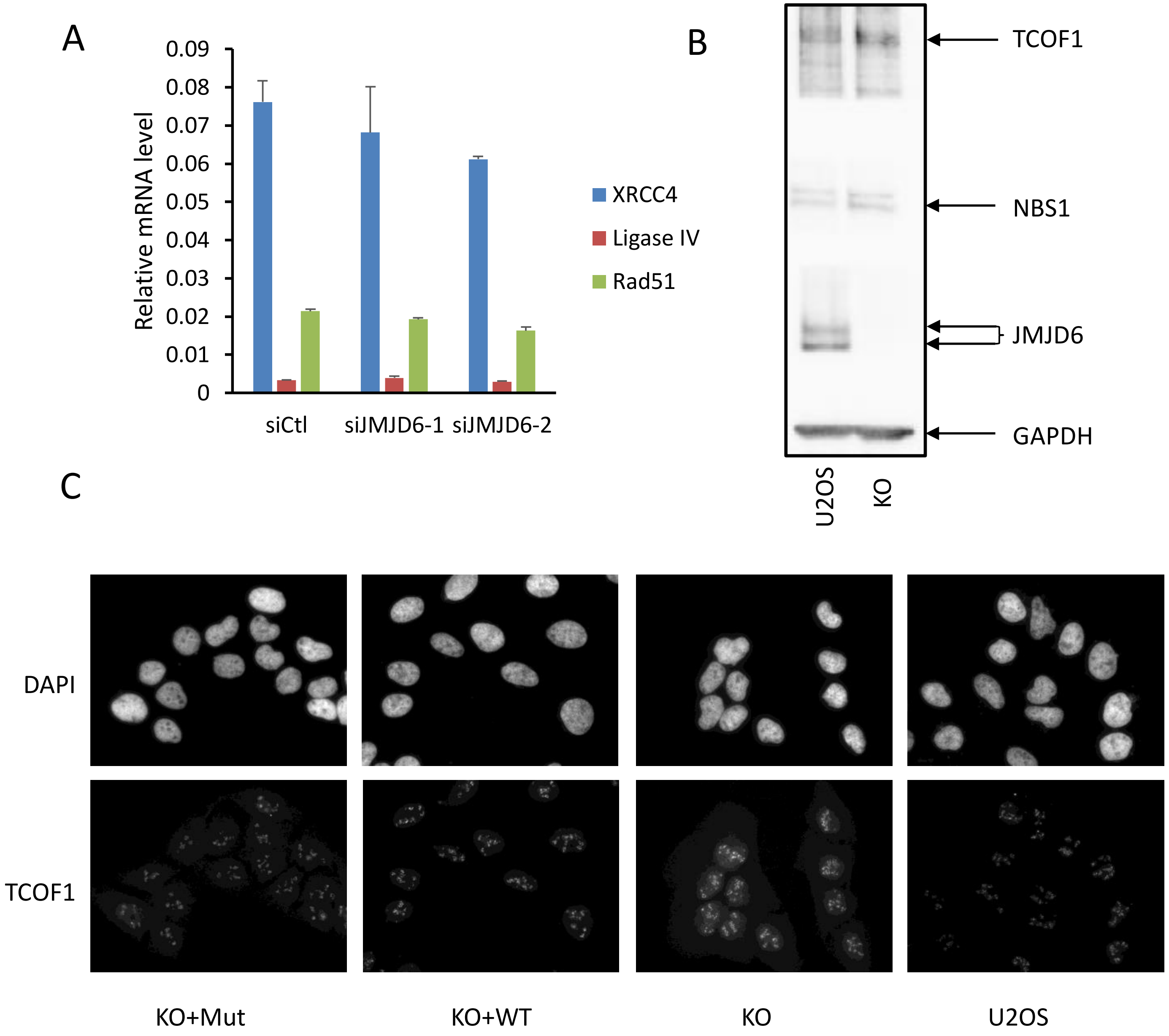

Fig S6. TCOF1 expression and localisation are not altered in JMJD6 KO cells.
(A) RT-qPCR performed for the evaluation of mRNA level of XRCC4, ligaseIV and Rad51 after JMJD6 depletion in U2OS cells. (B) Expression of TCOF1, NBS1 and JMJD6 in U2OS and JMJD6-KO cell lines assessed by Western blot. (C) Immunofluorescence against Treacle was performed in the different cell lines defective or not for JMJD6 expression.

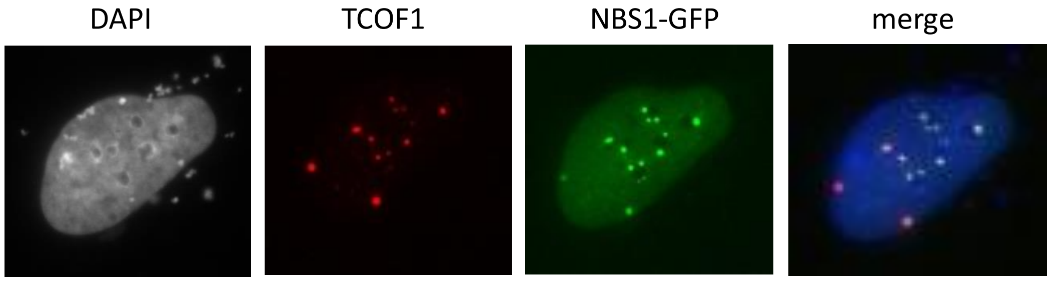

Fig S6D. TCOF1 and NBS1 co-localize in JMJD6 KO cells after rDNA-DSB induction.
JMJD6 KO cells were co-transfected with plasmids coding for NBS1-GFP and CRISPR-Cas9 inducing rDNA DSB. Immunofluorescence against TCOF1 was performed and co-localization with NBS1-GFP assessed.
