# Supplementary material for: JMJD6 participates in the maintenance of ribosomal DNA integrity in response to DNA damage
Source: PLoS Genet. 2020 Jun 29;16(6):e1008511. doi: 10.1371/journal.pgen.1008511 (PMC7351224; doi:10.1371/journal.pgen.1008511)
Supplement: S7 Fig — (PDF) [file pgen.1008511.s007.pdf]

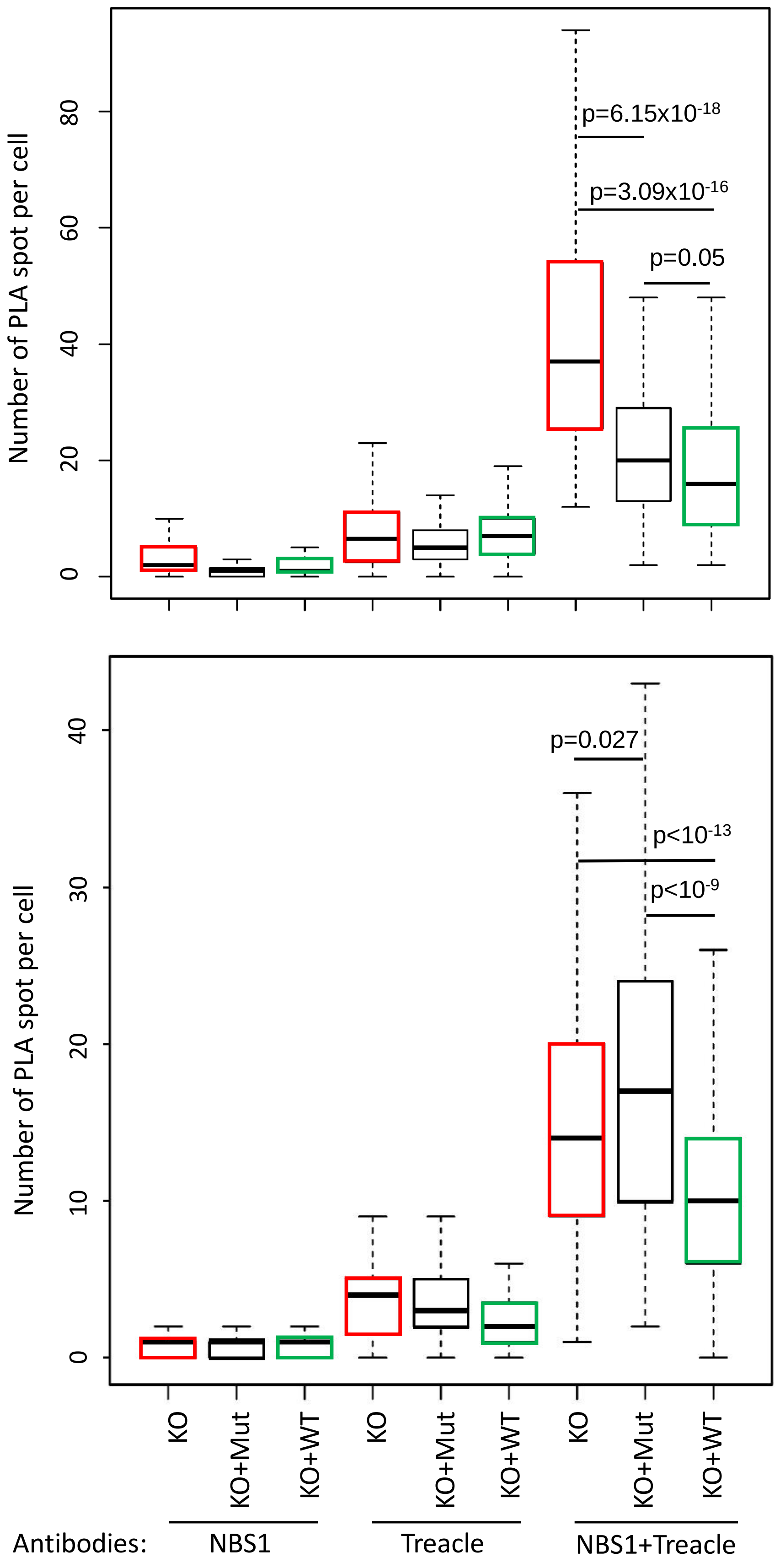

FigS 7. JMJD6 influences the NBS1-Treacle interaction.
Additional independent experiments from Figure 6 monitoring the NBS1-Treacle interaction by PLA after DNA damage (5 Gy 1h post-IR).
