# Supplementary material for: JMJD6 participates in the maintenance of ribosomal DNA integrity in response to DNA damage
Source: PLoS Genet. 2020 Jun 29;16(6):e1008511. doi: 10.1371/journal.pgen.1008511 (PMC7351224; doi:10.1371/journal.pgen.1008511)
Supplement: S8 Fig — (PDF) [file pgen.1008511.s008.pdf]

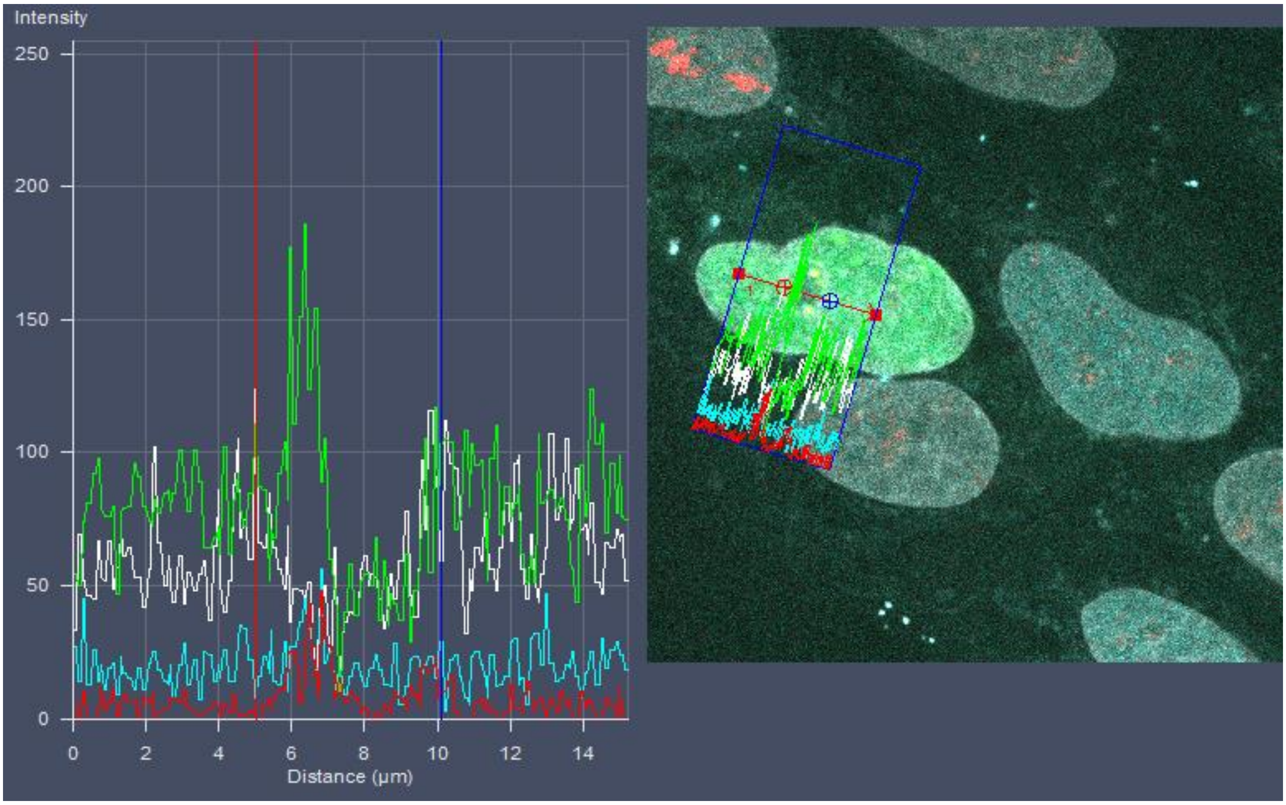

FigS8. JMJD6 colocalizes with Treacle and NBS1 in nucleolus.
(Right) Image of U2OS cells expressing V5-tagged-JMJD6 transfected with NBS1-GFP and exposed to ionizing radiations at 5 Gy (1 h post-IR) analysed by confocal microscopy using anti-V5 or anti-Treacle antibodies. (Left) Confocal line profile showing fluorescence intensity for Treacle (red), JMJD6-V5 (Cyan), NBS1-GFP (green) and DAPI (white). Note the correspondence of the peaks of Treacle and JMJD6 and NBS1 in DAPI low region corresponding to the nucleolus (between the red and blue vertical lines).
