# Supplementary material for: JMJD6 participates in the maintenance of ribosomal DNA integrity in response to DNA damage
Source: PLoS Genet. 2020 Jun 29;16(6):e1008511. doi: 10.1371/journal.pgen.1008511 (PMC7351224; doi:10.1371/journal.pgen.1008511)
Supplement: S9 Fig — (PDF) [file pgen.1008511.s009.pdf]

A

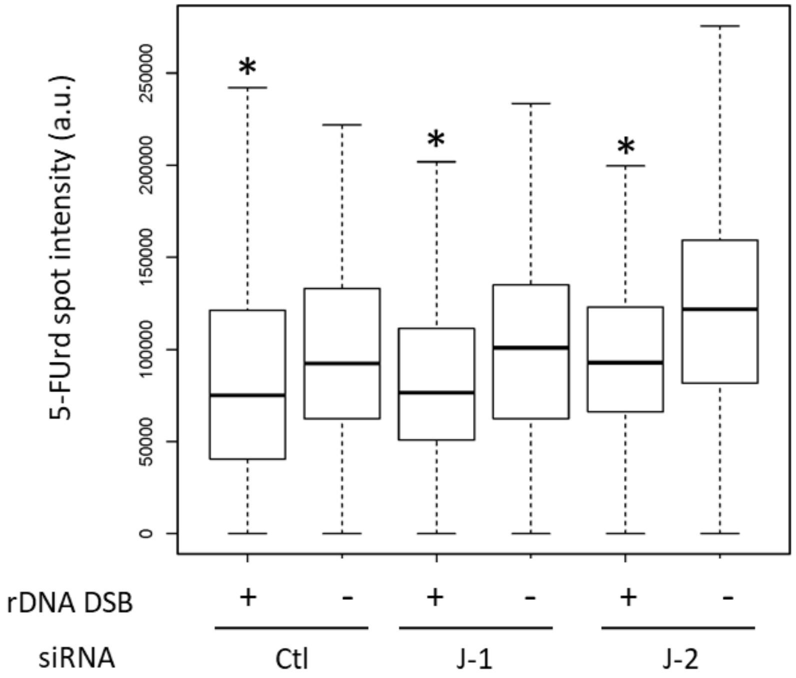

B

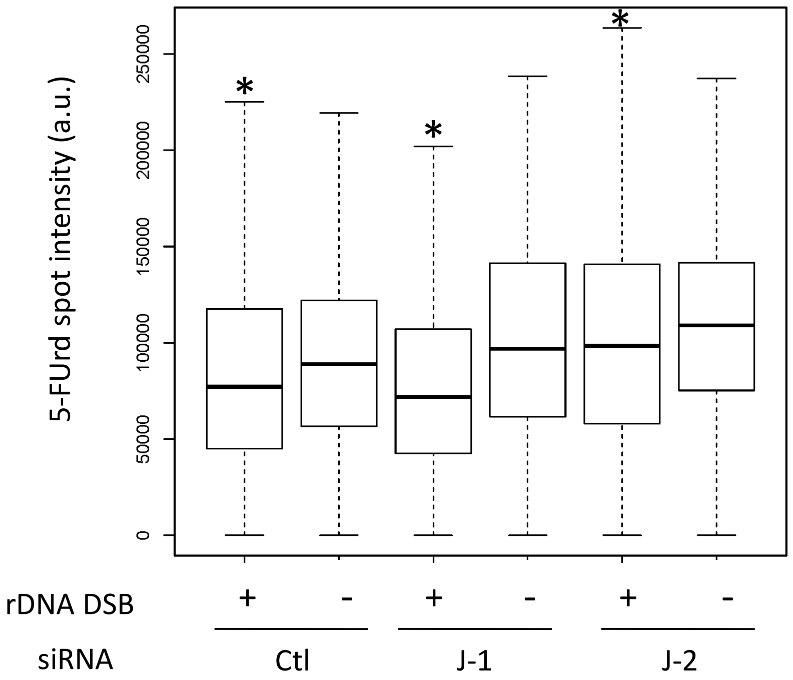

Fig S9. rDNA transcription in JMJD6-depleted MRC5 cells after rDNA DSB.
Specific rDNA DSB were induced using CRISPR-Cas9 in MRC5 cells depleted for JMJD6 Using siRNAs. 5-FUrd incorporation was detected by immunofluorescence after a 20 min pulse incorporation of 5-FUrd. In each point a minimum of 200 cells were analysed.
The p values for the difference between +/- rDNA DSB are : (A) $2.02.10^{-4}$; $3.95.10^{-4}$; $1.13.10^{-6}$ (B) $1.23.10^{-2}$; $1.25.10^{-9}$; $4.42.10^{-2}$ for siCtl, si J1 and si J2, respectively.
