# Supplementary material for: JMJD6 participates in the maintenance of ribosomal DNA integrity in response to DNA damage
Source: PLoS Genet. 2020 Jun 29;16(6):e1008511. doi: 10.1371/journal.pgen.1008511 (PMC7351224; doi:10.1371/journal.pgen.1008511)
Supplement: S10 Fig — (PDF) [file pgen.1008511.s010.pdf]

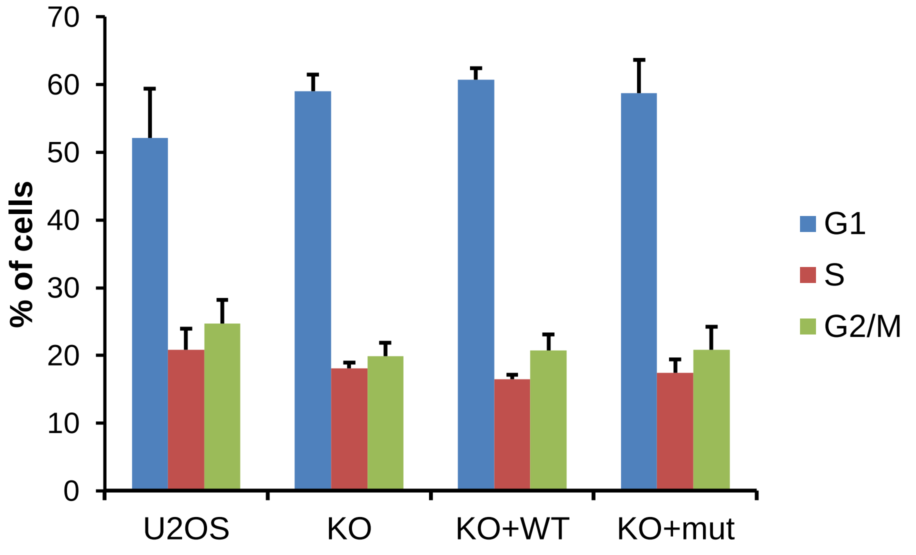

FigS10: cell cycle distribution of the JMJD6-KO cell lines and complemented cell lines.

The cells were ethanol-fixed, then stained with propidium iodide and analysed by flow cytometry. The repartition of the cells in the various phases of the cell cycle is indicated. Results are the mean +/- s.d. of 4 experiments.
