# Supplementary material for: JMJD6 participates in the maintenance of ribosomal DNA integrity in response to DNA damage
Source: PLoS Genet. 2020 Jun 29;16(6):e1008511. doi: 10.1371/journal.pgen.1008511 (PMC7351224; doi:10.1371/journal.pgen.1008511)
Supplement: S11 Fig — (PDF) [file pgen.1008511.s011.pdf]

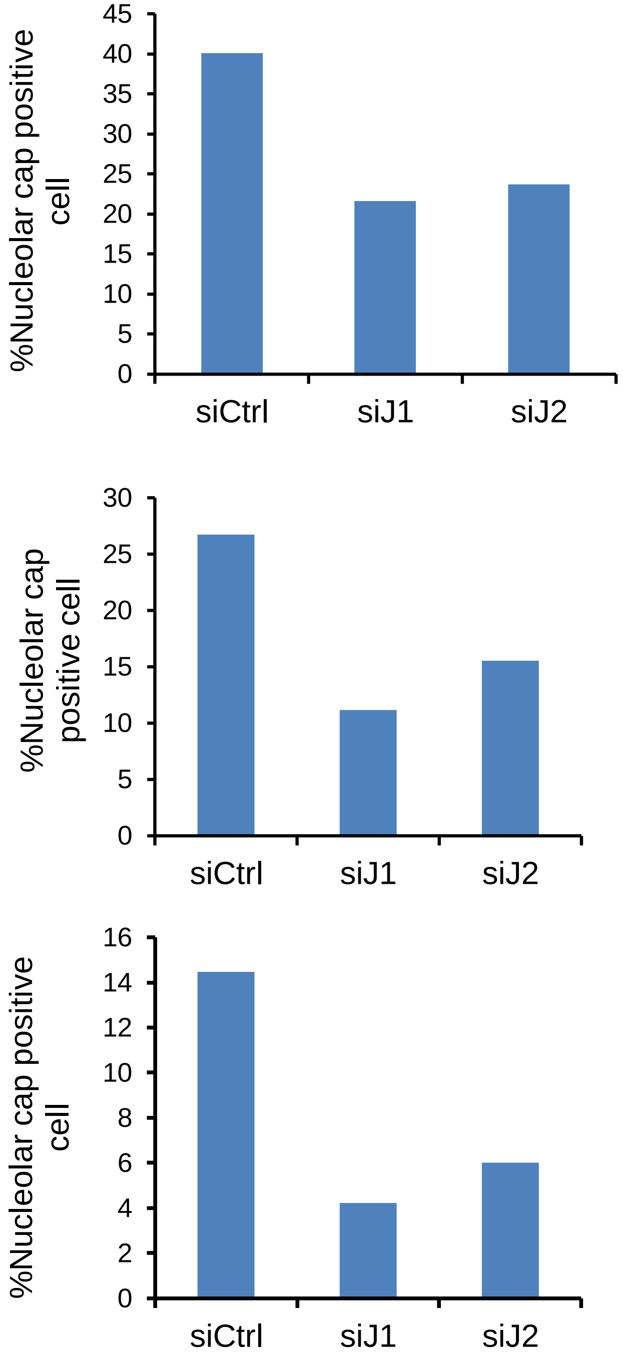

FigS11: **JMJD6 depletion affects nucleolar caps generation after ionizing radiations exposure.**
U2OS cells transfected with the indicated siRNA were irradiated (20 Gy) and fixed 6h post IR.
Quantification of nucleolar caps positive cells using UBF staining.
Three independent experiments are shown. For each point more than 200 cells were counted.
