# Supplementary material for: JMJD6 participates in the maintenance of ribosomal DNA integrity in response to DNA damage
Source: PLoS Genet. 2020 Jun 29;16(6):e1008511. doi: 10.1371/journal.pgen.1008511 (PMC7351224; doi:10.1371/journal.pgen.1008511)
Supplement: Attachment — Submitted filename: Review for manuscript by Fages et al.pdf [file pgen.1008511.s034.pdf]

Review for manuscript submitted to Plos Genetics, by Fages et al. entitled: ‘JMJD6 participates in the maintenance of ribosomal DNA integrity in response to DNA damage’ (PGENETICS-D-19-01818).

In this paper Fages et al. describe a role for JMJD6 in the response to DSBs occurring in ribosomal DNA (rDNA) repeats. The manuscript covers an important topic and contains some interesting findings. However, presentation of the results and quality of the data should be improved. Moreover, a number of issues about the role and significance of JMJD6 in the regulation of damage in the nucleoli remain to be addressed. The authors find that JMJD6 is involved in the response mediated by TCOF1-Nbs1 (Larsen et al. NCB 2014, Ciccia et al. PNAS 2014). However, JMJD6 is not required for this pathway’s main role; the repression of rDNA transcription. Instead the authors indicate that JMJD6 is important for the formation of nucleolar caps, and the recruitment of Nbs1 to the nucleolus. They speculate that loss these functions will influence repair of rDSBs. My biggest concerns is that the authors provide little evidence showing a role of JMJD6 in the repair of breaks in rDNA.

Main points:

- JMJD6 is a i) arginine demethylase, ii) lysyl hydroxylase and iii) tyrosine kinase. It is unclear which mutant was used in this study. The authors should provide a much more thorough functional-mechanistic explanations for the role of JMJD6. This could be easily addressed in the JMJD6 knockout cells (e.g. γH2AX foci-formation, sensitivity).
- It is unclear why cells without JMJD6 are sensitive to DSBs? How do the results in Figure 1 influence the DNA damage checkpoint? and do the observed γH2AX foci correspond to DSBs?
- JMJD6 is implicated in the regulation of transcription, mostly in the activation of genes. How does loss of JMJD6 influence transcription? Particularly of genes involved in the response to rDNA damage? Could loss of JMJD6 have an indirect effect?
- Experiments are performed in U2OS cells, can the authors exclude cell type-specific effects? Key experiments should be performed in another cell line, preferably in non-transformed cells.
- Does JMJD6 regulate DNA repair? Are IR sensitivity, UBF foci, rDNA rearrangements,… in JMJD6 knockout cells altered after the inhibition of DNA repair pathways? If JMJD6 is involved in promoting HR, would this result in more DSB resection and HR repair (RPA, Rad51 foci) and can the authors address this?

- Have the authors considered alternative models, for example a role for JMJD6 in the restart of transcription after damage in the rDNA. This would be consistent with JMJD6 known function in promoting transcription?

Minor points:

- Significance in the data plots should be indicated with stars (*) and the exact p-value mentioned in the legends.
- In some of the western blots shown, the lanes are separated with bars, as in the blot accompanying Figure 2A. Indicate (for all western blots in the manuscript) whether these lanes are from separate blots. If from separate blots, new blots should be generated containing all the indicated lysates from one experiment on one blot.
- The increase in γH2AX after knockdown of JMJD6 in Figure 1 should be repeated on western blot.
- The results showing laser-induced recruitment (e.g. Figure 1D, S1,..) should be quantified.
- What is the difference between results in Figure 2A, S3A and S3C?
- rDNA rearrangements experiment in Figure 3D should be explained more in depth.
- In Figure 3E the error-bars are missing.
- A wildtype (parental) should be included in Figure 4B and 5.
- Results in Figure 6 and 7 should be repeated in JMJD6 KO cells.
- What are the additional lower bands in the p-ATM blot after IR in Figure 8?
- In Figure S9 the error-bars are missing.
- The analysis of the images in Figure S6 and S8 is unclear and should be modified.
