# Supplementary material for: JMJD6 participates in the maintenance of ribosomal DNA integrity in response to DNA damage
Source: PLoS Genet. 2020 Jun 29;16(6):e1008511. doi: 10.1371/journal.pgen.1008511 (PMC7351224; doi:10.1371/journal.pgen.1008511)
Supplement: Attachment — Submitted filename: Response to Reviewers-final.docx [file pgen.1008511.s035.docx]

**Response to the reviewers comments:**

*Reviewer #2: Fages et al., identify JMJD6 as a protein involved in nucleolar DNA repair. At first the authors perform a screen to identify proteins, that upon depletion, elevate gH2AX levels after Ionising radiation. From this screen they select JMJD6 as a potential repair protein candidate for further investigation. Micro-laser irradiation reveals a strong consistent recruitment to nucleoli after DNA damage and a more moderate recruitment in nuclear chromatin. Clonogenic survival assay show increased sensitivity to nuclear and nucleolar DNA damage after depletion of JMJD6. rDNA instability is documented by decrease in mitotic UBF foci number and rearrangement of rDNA repeats. The authors then identify an interaction with the nucleolar protein Treacle/TCOF1 and that depletion of JMJD6 increases the interaction between Treacle and NBS1. The authors also find that transcriptional inhibition is not compromised in the absence of JMJD6. The authors then report that nucleolar cap formation is decreased in JMJD6 depleted cells and suggest that nucleolar cap formation is a process uncoupled from transcriptional inhibition. Finally the authors report less nucleolar NBS1 accumulation after IR in JMJD6 KO cells and propose that JMJD6 is responsible for NBS1 recruitment into nucleoli in an analogous way to Treacle/TCOF1. The data presented in this manuscript are interesting but in some places the experimental design makes it difficult to determine if the conclusions are valid. Furthermore, in some cases additional data is required to support the presented experiments.Specific comments for the authors*

We thank the reviewer for these very positive comments on our manuscript.

*Introduction:Page 5 line 4: When describing the nucleolar DNA damage response the novel discoveries of the involvements of ATR in nucleolar cap formation should be mentioned (Velichko et al., and Korsholm et al.,).*

We now quote this study and one additional performed by the group of Stucki showing the involvement of ATR in response to ribosomal DNA damage.

*Figure 2:Page 8 line 1: “Considering the size of the rDNA loci, the irradiation dose we used in the clonogenic cell survival assay should not lead to breaks in the rDNA in a significant proportion of cells”. The authors should explain how this was calculated and include references that documents this statement.*

We changed the manuscript to include this information and included references for the numbers of DSB induced by irradiation (ref 24) and the representation of the rDNA repeats among the total genomic DNA (ref 2).

*Figure 3EThe authors state “Results show that in absence of external DNA damage JMJD6 depletion per se induced a higher level of rDNA rearrangements (Figure 3E and Figure S5). In response to induced-DSB we observed an increase in rDNA rearrangements in control cells which was higher in JMJD6-depleted cells.” The data presented do not support the that induced-DSBs lead to more rearrangements in JMJD6 depleted cells compared to control cells. On the contrary the level of rearrangements induced by DSBs appears to be less or the same. The authors should provide a third experiment and test if the rearrangements induced with or without damage are significant.*

We performed a third experiment and statistical analysis as requested by the reviewer (Figure 4E of the revised manuscript).

*Figure 5:The authors report an increased interaction between NBS1 and TCOF1 in JMJD6-KO cells. Does this interaction occur in the nucleoli? The authors must co-stain with a nucleolar marker and quantify NBS1-TCOF1 PLA foci in the nucleus versus nucleoli to determine if this interaction is linked to nucleolar functions.*

Co-staining the nucleolus with a nucleolar marker is not so easy because we are already using 2 antibodies directed against 2 different species (mouse for Treacle and rabbit for NBS1) to perform the PLA assay. However, in order to confirm that the interaction between NBS1 and TCOF1 occurs mostly in the nucleolus we changed the blue DAPI images to black and white images and show a magnification of nucleoli region in insert (Figure 6).

*Figure 7:Page 12, the authors state “However, here we show that nucleolar caps formation and transcription inhibition can in fact be uncoupled since JMJD6 depleted cells display transcriptional repression, with yet less nucleolar caps.” If the authors want to claim uncoupling of nucleolar transcription and cap formation they must conduct experiments where these two read outs are measured at the same time with exposure to the same treatments. The current data is not consistent enough to make such claims.*

Actually, we were already using the same conditions in the DiVA cell line following induction of endonuclease-mediated DNA breaks (4 hours of treatment with OH tam to induce DSBs). These data show that in the same conditions, JMJD6 knock-down leads to defect in nucleolar cap formation while transcriptional repression is maintained. We now clarify that in the revised manuscript by adding details in the legends of figure 7 and 8 and in the text of the results section.

*Figure 9:The authors find that NBS1 recruitment to nucleoli is compromised in JMJD6 KO cells and assigns a role for JMJD6 in NBS1 recruitment. Previous studies, also cited in this paper, have shown Treacle/TCOF1 to be responsible for NBS1 recruitment into nucleoli. Furthermore, the authors have demonstrated that TCOF1-NBS1 interactions are increased in Figure 5 and it is unclear if this occurs in the nucleolus. An alternative explanation could therefore be an alteration in the level/localization of TCOF1 due to the significant amount of nuclear DNA damage generated in the absence of JMJD6. The authors should assay the total level of TCOF1 protein in the cell and the cellular localization of TCOF1 by IF to exclude that the observed changes in NBS1 recruitment is caused by altered localization of TCOF1. Finally the authors should test if NBS1 and TCOF1 co-localize in JMJD6 KO cells.*

We performed the requested experiment and observed no difference in TCOF1 expression and localization in JMJD6 KO vs complemented cells. In addition the co-localization of TCOF1 and NBS1 was evaluated in JMJD6 KO cells and both proteins co-localize. These data are now shown in supplemental Figure S5..

General comments:

Major concern:

*The changes and uncpecific action of the DNA damaging agents used throughout the manuscript makes it difficult to distinguish the nucleolar response from a general stress response activated in the nucleus. Absence of JMJD6 induces DNA damage genome-wide and the nuclear response to exogenous DNA damage is elevated upon treatment with IR. The authors repeatedly uses methods that activates multiple responses (DIvA endonuclease and IR) and a specific role for JMJD6 in the nucleolus therefore becomes hard to determine.*

As requested by the reviewer, in order to clarify the role of JMJD6 in response to nucleolar DNA damage we performed key experiments using CRISPR-Cas9 system with a guide targeting the rDNA. These data show that nucleolar cap formation induced by rDNA-specific breaks is defective in JMJD6 KO cells (Figure 8D) while transcriptional repression is maintained (Figure S11). We obtained similar results using an additional cell line, the MRC5 cell line (Figure 8E) : These new data are included in Figure 8 of the revised manuscript.

Minor comments:

*In all pictures, where relevant, the nucleolus should be indicated either by co-staining with a nucleolar marker or by an added line (generated in a computer software) following the edge of the nucleolus. Currently it is difficult to determine the nuclear localization of features such as caps and foci in the included images and therefore it is difficult to judge their link to nucleolar functions.The dosis of IR should be stated in all the figure legends.*

We modified the text accordingly and we now indicate the nucleolus with a dash line on color images or we replaced the blue DAPI images by a grey images to increase contrast of DAPI staining.

Reviewer #3:

We thank the reviewer for the very positive comments on our manuscript in the introduction paragraph.

*Figure 1. Correct the labelling and legend of Fig 1. Legend indicates that part A is the western, part B is the IF images and C is the quantification. Why does siJMJD6-2 not produce a similar result to siJMJD6-1 in the γH2AX IF, despite the quantification suggesting even greater γH2AX intensity? Correct or comment. Also comment that the nuclear and nucleolar striping of JMJD6-GFP in supplemental Figure S1C have similar timing indicating that recruitment to these two compartments is coincident in those cells that display JMJD6-GFP localisation to laser stipes in both compartments. Could this be an artefact of fluctuations of laser power between different experiments or can this result be seen in different cells from the same experiment?*

We thank the reviewer for having spotted this. Indeed, Figure 1 was actually misleading with an apparent weak signal in the siJMJD2 sample. This is due to automatic settings when exporting the images from the device allowing automatic analysis of fluorescence (two strong spots in this field led to an apparent decrease of the signal). Images are shown as examples, and the interpretable result comes from the quantification. We changed this image to avoid confusion.

We also changed the text according to this reviewer suggestion.

*As the authors failed to see increased recruitment of JMJD6 via ChIP (Fig S2), confirmation of JMJD6 recruitment to nucleolar-specific DSBs induced via CRISPR/Cas9 should be attempted to resolve the issue of whether this protein is actually recruited to DSBs, i.e. confirm the laser striping result, especially as this technique is prone to artefact.*

We performed experiments in which we co-transfected JMJD6-GFP constructs together with CRISPR-Cas9 targeting the rDNA. Results show that in some case it is possible to observe JMJD6-GFP recruitment in the nucleolus. However, this is not observed in all cases certainly due to the kinetics we used (16 h post transfection) because the phenomenon is very rapid in living cells after laser striping (around 1-2 min). Examples of JMJD6-GFP recruitment observed in the nucleolus are now shown in figure S2 with the previous ChIP experiment.

*Figure 3E. A third experiment with proper quantification and statistical significance should be included. This is important as the effect sizes are relatively small. Also, Fig S5. Would a knock out give better results than the two knock downs used?*

We performed the requested experiment and now present statistical analysis made on the 3 experiments (Figure 4E of revised manuscript). Figure S5 has been removed.

*Figure 4. For the convenience of the reader, the complete results of the proteomic screen should be provided in a supplemental table, as well as in the MassIVE database. Validation of the JMJD6 interaction with Treacle by PLA and co-localisation is insufficient. The authors should also use another biochemical approach, e.g. co-IP. Also, is this interaction direct (detectable with recombinant proteins) or indirect? The legend for Figure 4 require more information about the experiment shown to facilitate the reader.*

We now validated the interaction by co-IP in U2OS cells and by Bio-ID in HEK293 cells. These data are shown in Figure 5 and Table S1 and S2 of the revised manuscript. In the time allowed for revision, we did not manage to investigate the potential direct interaction between JMJD6 and Treacle with recombinant proteins. However, the data obtained with the Bio-ID experiments are considered to give information on proteins on close vicinity (10 nm) that can be considered as direct as in the case of FRET experiments.

*Figure 5. Again the effects of JMJD6 on the Treacle-NBS1 interaction must be confirmed biochemically (e.g. co-IP) as the PLA assay is insufficient for the conclusions drawn. A third repeat with proper quantification is also required, especially important given the relatively small effect sizes shown. The legend to his figure also requires more information to facilitate the reader.*

We have now performed four independent experiments and obtained similar results in all of them. A third experiment is now presented in Figure S7. We now specify that in the revised manuscript. We tried to confirm this result by co-immunoprecipitation as proposed by this reviewer, but in the time allowed for revision, these experiments were inconclusive. We decided to keep the PLA data given that they are reproducible and robust, but toned down the conclusion in the revised manuscript.

*Figure 6. As noted above, there is confusion in the manuscript between an observation noted as: “strikingly, rDNA transcription was further decreased in JMJD6-depleted cells…” and their interpretation of no effect on the rDNA transcriptional response. Why did the authors not use the more specific CRISPR/Cas9 approach, as used in Figure 2, to induced DSBs specifically in rDNA repeats? As this would be free of any confounding effects of DSBs elsewhere in the nucleolus it may well give more specific results with perhaps greater effect sizes. While I think the data is clear that JMJD6 does not have a large role in the rDNA transcriptional role to DNA damage, if it does modulate the Treacle-NBS1 interaction it might be expected to modulate this transcriptional response. Can the authors reassess the possible role of JMJD6 in the rDNA transcriptional response to DNA damage specific to the rDNA repeats? They have this capability as demonstrated in Figure 2 and with the addition of a transfection control this approach should be possible. Once more the JMJD6-specific effect sizes of the experiments shown are, unfortunately, small (certainly not striking!). Therefore, quantification of three separate experiments should be combined and analysed for significance as a combined data set. Also, use the JMJD6 knockout rather than siRNA-mediated depletion. The legend to his figure too requires more experimental information to facilitate the reader. Replace “OHT” with “AsiSI”.*

We performed the requested experiment using CRISPR-Cas9 as specific inducer of DNA DSB in rDNA and found that transcriptional repression is still observed in an additional cell line, the MRC5 cells depleted for JMJD6 using siRNAs. This result is now presented in the figure S11.

In the new figure 7 we replaced "OHT" with "AsiSI" as requested by the reviewer.

*Figure 7. This is a convincing result suggesting uncoupling of rDNA transcriptional repression from nucleolar cap formation. However, it would be more convincing to include an experiment using the CRISPR/Cas9-induced rDNA-specific DSBs. Note Figure S10, using IR rather than AsiSI, is missing from the downloaded PDF.*

We performed the requested experiment which is now shown in Figure 8. We performed experiments using CRISPR/cas9 induced rDNA-specific DSB in the JMJD6 KO and in a non transformed cell line, MRC5, depleted for JMJD6 by using siRNAs. In both case we observed a defect in the generation of nucleolar caps in response to rDNA induced DSB in JMJD6 deficient cells. These results are now presented in the figure 8 to reinforce and confirm the role of JMJD6 in the generation of nucleolar caps in response to DNA damage occurring on rDNA.

Figure S10 was missing, and we apologize for this mistake.*.*

*Figure 8. If JMJD6 is specific to the nucleolar DDR using IR is a poor choice of DSB-inducing agent for these experiments and should be repeated with rDNA-specific DSBs.*

In the time allowed for revision, we tried to perform the requested experiment but results were inconclusive. Given that ATM is a known mediator of DSB signaling in the nucleolus after DNA damage induced at rDNA (van Sluis and McStay, 2015), we concentrated our work on others points central to our message.

*Figure 9. In part A, the images are not convincing, as shown, with respect to NBS-GFP foci being actually in a nucleolus. It would be much better resolution to show both the DAPI and GFP channels in black and white (similarly for other Figures), rather than false coloured green and blue. Once more, repeating with rDNA-specific DSBs would add significantly to this figure and increase confidence in the overall result.*

We modified the Figure as suggested by the reviewer for the color of the different channels. We performed the requested experiment. However, we found that in these conditions, 100% of transfected cells have nucleolar caps with NBS1-GFP, probably as a result of the continuous induction of plenty of DSBs in the nucleolus through a guide targeting a repeated sequence and the overexpression of NBS1. As a consequence, and given that, as we state in the manuscript, JMJD6 participates in but is not completely required for nucleolar cap formations, no major difference could be observed between control and KO cells. These data were not included in the revised manuscript.

Reviewer #1

*Review for manuscript submitted to Plos Genetics, by Fages et al. entitled: ‘JMJD6 participates in the maintenance of ribosomal DNA integrity in response to DNA damage’ (PGENETICS-D-19-01818).*

*In this paper Fages et al. describe a role for JMJD6 in the response to DSBs occurring in ribosomal DNA (rDNA) repeats. The manuscript covers an important topic and contains some interesting findings. However, presentation of the results and quality of the data should be improved. Moreover, a number of issues about the role and significance of JMJD6 in the regulation of damage in the nucleoli remain to be addressed. The authors find that JMJD6 is involved in the response mediated by TCOF1-Nbs1 (Larsen et al. NCB 2014, Ciccia et al. PNAS 2014). However, JMJD6 is not required for this pathway’s main role; the repression of rDNA transcription. Instead the authors indicate that JMJD6 is important for the formation of nucleolar caps, and the recruitment of Nbs1 to the nucleolus. They speculate that loss these functions will influence repair of rDSBs. My biggest concerns is that the authors provide little evidence showing a role of JMJD6 in the repair of breaks in rDNA.*

We thank the reviewer for these positive comments on our manuscript. We would like to point that, although we acknowledge that we do not fully understand the function of JMJD6 in the repair of the rDNA, we provide many clues from independent experiment settings indicating that this function is important:

- We show that JMJD6-depleted cells are more sensitive to DNA breaks occurring specifically in the rDNA (Fig 3).
- We show that JMJD6-depleted cells harbour higher genetic rearrangements at rDNA repeats (Fig 4).
- We show that JMJD6-depleted cells have defects in nucleolar cap formation (Fig 8), which are considered as structures important for repair of rDNA breaks. Furthermore, in the revised manuscript, we show that these defects are also observed when breaks are induced only in the rDNA.
- We show that JMJD6-depleted cells have a defective nucleolar dynamics of NBS1, which is important for the repair of rDNA breaks (Fig 6 and 10D).

*Main points:*

*- JMJD6 is a i) arginine demethylase, ii) lysyl hydroxylase and iii) tyrosine kinase. It is unclear which mutant was used in this study. The authors should provide a much more thorough functional-mechanistic explanations for the role of JMJD6. This could be easily addressed in the JMJD6 knockout cells (e.g. γH2AX foci-formation, sensitivity).*

We now describe better the mutant used in this study. This mutant harbors 3 point mutations in the catalytic domain of JMJD6 preventing Fe(II) binding (Chang et al 2007 Science 318: 444-447). This mutant disrupts both the hydroxylation and demethylase activity, given that hydroxylation is the first step towards demethylation. It is thus impossible to distinguish between these two activities using point mutants (Note however that whether JMJD6 possesses arginine demethylase activity is highly debated in the field).

To address this point, we thus analysed methylation of R2 from histone H3, which is a described substrate of JMJD6 demethylase activity. By ChIP experiments, we found that the level of H3R2me methylation is low in the rDNA and is not changed upon breaks induction. In addition, its level is roughly similar in JMJD6 KO cells (data not shown). So we could not find any evidence of an involvement of the described demethylase activity of JMJD6 in rDNA breaks management.

As stated by the reviewer, JMJD6 has also been described as a tyrosine kinase (Liu et al 2018). However, in this study, the mutant they used as a kinase-dead mutant harbours a deletion of the JMJC domain and the poly(S) domain at the C terminus of JMJD6. It is thus also defective for lysyl hydroxylase and arginine demethylase activity, precluding its use to analyse the specific function of the tyrosine kinase activity.

We changed the text of the results section to specify this important point.

*- It is unclear why cells without JMJD6 are sensitive to DSBs? How do the results in Figure 1 influence the DNA damage checkpoint? and do the observed γH2AX foci correspond to DSBs?*

As shown later in the text, ATM activation is not affected by JMJD6 depletion. In addition, we now performed experiments using reporter systems addressing the function of JMJD6 in DSB repair and found that JMJD6 expression is influencing NHEJ but dispensable for Homologous Recombination. These data are included in the revised manuscript as Figure 2. We can speculate that inefficient repair of DSB in the absence of JMJD6 is the reason of the higher sensitivity of JMJD6-depleted cells to ionizing radiations.

We agree with the reviewer that we don’t know whether the observed γH2A.X foci corresponds to DSBs. γH2A.X is a widely used marker of DSBs. However, it is true that it can marks other DNA damage than DSBs, and this is why in the manuscript we are mostly using other ways to induce DNA damage, which specifically produce DSB, such as CRISPR-Cas 9 or endonuclease (AsiSI). Note that we observe hypersensitivity of JMJD6-depleted cells upon specific induction of DSBs in the nucleolus (Figure 3).

*- JMJD6 is implicated in the regulation of transcription, mostly in the activation of genes. How does loss of JMJD6 influence transcription? Particularly of genes involved in the response to rDNA damage? Could loss of JMJD6 have an indirect effect?*

We checked the expression of a number of mRNAs coding for proteins involved in DNA breaks management in the nucleolus and did not find any effect of JMJD6 depletion. We tested XRCC4, DNA ligase IV and Rad51 expression using RT-qPCR. These data have been added in the revised manuscript in Figure S5. In addition, we checked the expression of identified essential proteins involved in the response to rDNA such as TCOF1 and NBS1 and observed no effect of JMJD6 depletion on the expression of such proteins (see Figure S5).

*- Experiments are performed in U2OS cells, can the authors exclude cell type-specific effects? Key experiments should be performed in another cell line, preferably in non-transformed cells.*

We now present data in MRC5 cells, which are not tumoral. MRC5 is a normal embryonic fibroblast cell line derived from lung (ref ATCC CCL171) and immortalized using SV40 T antigen. We confirmed in this cell line the most important results of our study, ie that JMJD6 is important for nucleolar caps formation. These data are now shown in Figure 8E of the manuscript. In addition we also observed that JMJD6-GFP is recruited at laser stripes in the nucleolus of MRC5 cells (Figure S1D) and that, as in U2OS cells, siRNA depletion of JMJD6 does not prevent the transcription inhibition of the rDNA in response to targeted rDNA DSB (Figure S11).

Finally, we show that the TCOF-JMJD6 interaction is observed also in K562 cells (after affinity purification and mass spectrometry) and in HEK293 cells (by Bio-ID), indicating that this interaction is rather conserved. These data are now included in Figure 5 of the revised manuscript.

- *Does JMJD6 regulate DNA repair? Are IR sensitivity, UBF foci, rDNA rearrangements,… in JMJD6 knockout cells altered after the inhibition of DNA repair pathways? If JMJD6 is involved in promoting HR, would this result in more DSB resection and HR repair (RPA, Rad51 foci) and can the authors address this?*

To analyse the function of JMJD6 in DNA repair, we assessed the effect of JMJD6 depletion on the 2 mains DSB repair pathways, NHEJ and HR using cell lines harboring specific DNA repair substrate. We observed that JMJD6 depletion affected DSB repair by NHEJ while no detectable effect was observed on the repair of DSB by Homologous recombination. However, it has to be kept in mind that such assessment is performed in cell lines in which the DNA repair reporter system is not integrated in ribosomal DNA. The data showing the effect of JMJD6 depletion on repair of DSB are now included in Figure 2.

In the time allowed during revision, we were not able to perform the other requested experiments. However, we were showing in the original manuscript data indicating that, at least for nucleolar caps formation (which is the main process linked to rDNA breaks repair affected by JMJD6 depletion), inhibition of ATM (the main sensor kinase involved in rDNA breaks management) does not further decrease nucleolar caps formation in JMJD6 depleted cells. These data show that there is an epistatic relationship between JMJD6 and DNA repair pathways.

*- Have the authors considered alternative models, for example a role for JMJD6 in the restart of transcription after damage in the rDNA. This would be consistent with JMJD6 known function in promoting transcription?*

The reviewer is right in pointing this interesting possibility. Actually, we were already discussing this in the original manuscript. We now extend this discussion. Note however, that restart of rDNA transcription seems normal 6 hours following irradiation in JMJD6 knock-down cells (Figure 7).

*Minor points:*

*- Significance in the data plots should be indicated with stars (*) and the exact p-value mentioned in the legends.*

We decided not to follow this suggestion when numbers can easily be easily read in order to keep figure legends as simple as possible. However, we followed this reviewer' suggestion when Figures are more complicated, such as new Figure 4E and S11.

- *In some of the western blots shown, the lanes are separated with bars, as in the blot accompanying Figure 2A. Indicate (for all western blots in the manuscript) whether these lanes are from separate blots. If from separate blots, new blots should be generated containing all the indicated lysates from one experiment on one blot.*

The data are never from separate blots. The lines only indicate cuts that were performed to suppress lanes irrelevant for the message we draw from the blot. We now specify that in the figure legends.

*- The increase in γH2AX after knockdown of JMJD6 in Figure 1 should be repeated on western blot.*

We do not have any longer access to a gamma-Ray irradiator, so that we cannot easily repeat this experiment. However, γH2A.X foci observed by immunofluorescence are widely used markers for double strand breaks. Given that this experiment is the first of the paper, we believe that this IF is sufficient to introduce the question of the function of JMJD6 in DNA breaks management.

*- The results showing laser-induced recruitment (e.g. Figure 1D, S1,..) should be quantified.*

This is now done and is presented in the figure S1 of the revised manuscript.

*- What is the difference between results in Figure 2A, S3A and S3C?*

Fgures S3A and S3C are included to show that similar effect were obtained in two separate clones and are not the consequences of off target effects of CRISPR/cas9 made KO cell lines. This is now specified in the manuscript.

*- rDNA rearrangements experiment in Figure 3D should be explained more in depth.*

We changed the Figure legend in the revised manuscript according to this reviewer's suggestion.

*- In Figure 3E the error-bars are missing.*

Errors bars have been added with statistics after the inclusion of the third experiment.

*- A wildtype (parental) should be included in Figure 4B and 5.*

Using the parental cells in Figure 4B will not add anything to the use of the KO cells, given that JMJD6 is not tagged by V5 in parental cells, so will give background as in KO cells. In the time allowed for revision, we were not able to repeat Figure 5 experiments, which are rather lengthy to analyse, with parental cells.

*- Results in Figure 6 and 7 should be repeated in JMJD6 KO cells.*

Concerning Figure 6, rDNA transcription assay was performed in MRC5 cells depleted for JMJD6 using siRNAs in order to confirm that the observed effect was not only specific of the U2OS cell line used. This result is presented in Figure S11.

Concerning Figure 7, we analysed nucleolar caps formation in JMJD6 KO cells upon generation of DSB in the nucleolus using CRISPR-cas9 targeting the rDNA. These data confirmed the defect in the generation of nucleolar caps observed in JMJD6- siRNA depleted cells. In addition, the same experiment was performed using siRNA in a cell line not cancer- derived, MRC5. The results are presented in Figure 8 of revised manuscript as panels 8D and E.

*- What are the additional lower bands in the p-ATM blot after IR in Figure 8?*

These bands are probably shorter forms of P-ATM since they are induced by irradiation. This is now stated in the figure legend.

- *In Figure S9 the error-bars are missing.*

To reinforce the data of this figure, the cell cycle analysis has been performed in the different KO and complemented cell lines. The experiment has been performed 4 times and the error-bars have been added.

*- The analysis of the images in Figure S6 and S8 is unclear and should be modified.*

We modified the figure legends to clarify this analysis, as requested by the reviewer.
