# Supplementary material for: JMJD6 participates in the maintenance of ribosomal DNA integrity in response to DNA damage
Source: PLoS Genet. 2020 Jun 29;16(6):e1008511. doi: 10.1371/journal.pgen.1008511 (PMC7351224; doi:10.1371/journal.pgen.1008511)
Supplement: Attachment — Submitted filename: Reponse to the reviewers-23-04.docx [file pgen.1008511.s036.docx]

**Responses to the review comments**

**Comments to the Authors:**

*Reviewer #1:*

*The additional experiments performed by the authors have improved the quality of manuscript, as it now contains supportive evidence for the most important observations.*

We thank the reviewer for these positive comments.

*However, the authors should provide further explanation and insight in the discussion on the results with the catalytic mutant (Fig 3A, S4, 6 and S7), which seem contradicting.*

We have included a paragraph in the discussion to propose a speculative mechanism

to address this point (as well as a concern from reviewer 2). Page 17 line 11 of the final marked up copy. Page 16 line 11 of the clean copy.

*Although little mechanistic insight is revealed an explanatory model could be helpful for readers. Also, for the sake of the reader, further improvements to the outline of the manuscript and formatting of the figures are still urgently advised.*

We have added a model in a new Figure 11 and carefully reviewed the manuscript to improve the text and the presentation of the figures. In particular we modified Fig1 and Fig2 by moving the panel 1D to Fig 2 in order to clarify the text. We also inverted citations of Fig S5 and S6.

*Reviewer #2:*

*The manuscript by Fages et al. has improved as a result of several new data panels and text edits. One of the major points of interest, the uncoupling of transcriptional inhibition and cap formation, has also been strengthened in the current version of the manuscript. However, a few points still need to be addressed as outlined below.*

We thank the reviewer for these positive comments.

Comments to the authors:

“We also examined JMJD6-GFP recruitment after specific induction of rDNA DSB using CRISPR-Cas9. We could observe some cells, but not all (around 15%), harbouring punctuate accumulation of JMJD6-GFP in the nucleolus in agreement with a rapid and transient recruitment of JMJD6 at rDNA breaks (Fig S2).”

These data are inconclusive as they are. The presented panel in figure S2 should include enlargements of all channels presented above, not only GFP. Furthermore, the 15% of cells with JMJD6 in nucleoli are likely to be caused by the transient expression of JMJD6.Therefore a proper control should be included, either a non-targeting gRNA and/or an catalytic dead version of Cas9 and the number of cells with JMJD6 positive nucleoli should then be quantified and compared.

The panel presented in FigS2 has been removed because it was not possible for us to perform experiments with all the controls requested by the reviewer in a reasonable time. The reason is the present situation (containment because of the covid-19 pandemia) that does not allow access to the laboratory. However, we think that the absence of such experiment does not preclude the validity of any of our conclusions.

*"Considering the size of the rDNA loci (representing approximately 0.5% of the genomic DNA, from the calculation made by considering that the human genome is composed of three billions base pairs, that one rDNA repeat is approximately 40 kb in size and that there is around 300 rDNA repeats [2]), the irradiation dose we used (a 1 Gy dose generates approximately 20-40 DSB [24]) in the clonogenic cell survival assay should not lead to breaks in the rDNA in a significant proportion of cells."

Is this simple calculation valid? It appears very controversial and to simplistic. Nucleoli occupies a large area of the human nucleus and number of basepairs is likely not a determinant for the area a genomic domain occupies and therefore nor the likelihood of IR to induce a DSB. The authors should remove this argument or provide direct evidence.*

We removed this argument as suggested by the reviewer.

*"Taken together, this data indicates that the full expression of JMJD6 is specifically required for the management of DSBs occurring at the rDNA locus, consistent with our finding that JMJD6 is always recruited to the nucleolus upon DNA damage."

The last sentence should be toned down as it does not reflect the included data*

*accurately.*

We toned down the last sentence (Page 10 line 3) of the marked up copy. Page 9 line 4 of the clean copy. It is now written:

these data indicate that the full expression of JMJD6 is specifically required for the management of DSBs occurring at the rDNA locus, consistent with our finding that JMJD6 can be recruited to the nucleolus upon DNA damage.

*"More importantly, these results were confirmed by using CRISPR-Cas9 system targeting DSB at rDNA (Fig 8D and E), indicating that it is not due to a signalisation from DNA breaks induced outside the nucleolus. JMJD6 depletion by siRNA also affected nucleolar caps produced upon induction of breaks in the rDNA in the immortalized MRC5 cell line, indicating that it is not restricted to U2OS cells. (Fig 8E)."

The authors should include representative images showing the decrease in nucleolar caps in U2OS and MRC5 along with the graphs currently included.*

Representative images of nucleolar caps formation after induction of rDNA DSB in U2OS and MRC5 cells have been added in the Figure 8.

*"Using the JMJD6-KO cell lines and the WT or Mutant -complemented cell lines, we observed a higher level of interaction between NBS1 and Treacle in JMJD6 KO and Mutant-complemented cell lines compared to WT-complemented cell line after IR exposure (Fig 6 and Fig S7)."

And then….

"As expected, we observed an increase in NBS1 foci in the nucleolus of control cells in response to IR exposure. In contrast, IR-induced nucleolar localization of NBS1 was strongly decreased in JMJD6 KO cells, an effect which was reversed when these cells were complemented with JMJD6 but not in the cells complemented with the inactive form of JMJD6 (Fig 10B)………… These results show that JMJD6 and its enzymatic activity are required for the recruitment of NBS1 to the nucleolus in response to DNA damage, providing a direct link between JMJD6 and the presence of DNA repair factors in the nucleolus."

The data presented is contradictory and the authors need to provide an explanation for the results and what the differences between the results are.*

The reviewer is right in pointing this out. We have included a paragraph in the discussion section to propose a model (Fig 11), although speculative, reconciling these two apparently contradictory findings. Additionally, this model also allows us to discuss a point raised by reviewer 1. Paragraph added on page 17 line 11 of the final marked up copy. Page 16 line 11 of the clean copy.


Minor comment:

rDNA; ribosomal RNA genes

*Reviewer #3: While the authors have largely addressed most of my concerns, they were not able to address all concerns within the time allowed for resubmission. It is appreciated that the authors attempted to confirm the effects of JMJD6 on the Treacle-NBS1 integration by co-IP, a technique that is often troublesome. While this is still a weakness of the study, the revised study is much improved and has sufficient novelty to be of interest to those interested in the nucleolar DDR. Over all, my concerns have be adequately addressed.*

We thank the reviewer for these positive comments.
